# News and Notes

**Published:** 1899-10

**Authors:** 


### *NEWS AND NOTES.*

Dr. Dunbar Roy has been elected associate member of the American Ophthalmological Society.

An exchange asks, "is life worth living"? This question has been answered before. It depends upon the liver.

Professor Charles Stoerk, the eminent laryngologist, died in Vienna on September 14th.

The prospects for an increased number of students at the Atlanta College of Physicians and Surgeons are very flattering. Atlanta offers to students exceptional clinical advantages.

### Professional Dignity.

Full-bodied, awesome, solemn, grave, and wise,
Struts dignity with stately tread and slow.
A slip—a fall—and lo! before the eyes
The empty, leering mask of Pierrot.

But no Remedy.—Doctor: "Have you taken any remedy for this trouble?"

Patient: "No, doctor, I have not; but I've taken a power of medicine."—*Harlem Life.*

Creditors Uneasy Too.—Briggs: "It makes me uneasy to owe a cent."

Griggs: "I'm glad that I don't feel that way."

Briggs: "Why?"

Griggs: "Why? I'd have St. Vitus's dance."—*Puck.*

THE attempt of the California State Board of Health to establish a quarantine against human beings and domestic animals suffering with tuberculosis is not likely to be imitated elsewhere. The quick-witted inhabitants of Arizona have already issued a statement that the climate of that State is so dry and pure that there can be no reason whatever to fear the spread of tuberculosis there.

---

PROFESSIONAL CONVENIENCE.—Patient: "I say, doctor, just what is this 'grip' anyway?"

Doctor: "Why, my good fellow, that's the name we doctors have for everything nowadays but appendicitis."

Patient: "Ah! and what is appendicitis?"

Doctor: "Why, that's the name we have for everything but the 'grip.'"—*Judge.*

---

THE physicians in Wheeling, W. Va., have undertaken a crusade against all who practice without a degree. At a special meeting of the State Medical Society a committee was appointed to raise funds for this object. Not only will Christian Scientists and other charlatans be prosecuted, but an effort will be made to correct abuses arising under the old State laws by which licenses are granted to unqualified and often ignorant persons.

---

THE struggle to determine the position of women in Germany continues unabated. After the students at Halle protested against the admission of women to the medical courses of that university, the Woman's League of Berlin submitted an address to the Federal Council of the Empire asking for the full admission of women to the medical departments of the universities and to the examinations in medicine. So far as the Universities of Giessen and Strasburg are concerned, this has already been granted. The government of the Grand Duchy of Baden is equally pronounced in their favor. In Berlin the administrators of orphanages have employed women in certain offices. On the other hand, the administration of the railways has just decided that any woman employed in connection with them shall receive from $25 to $50 less than that received by men for the same work.

---

EDWIN G. DEXTER writes an article on the subject of "Influence of the Weather upon Crime" in the *Popular Science Monthly* for September, and concludes that the tendency to homicide varies with those meteorological conditions which bring about an emotional state necessitating a considerable discharge of motor stimulus. The same conditions bring about irritability and unruliness in the child, accompanying suicidal tendencies. This supposition is upheld by the fact that suicide is less common in the colder climates where the metabolic processes are slow, and in the torrid zone where the heat produces a general depletion of energy for motor discharge, than in the temperate regions where the climate is exhilarating.

